# Compact Fitting Formulas for Electron-Impact Cross Sections

**DOI:** 10.6028/jres.097.032

**Published:** 1992

**Authors:** Yong-Ki Kim

**Affiliations:** National Institute of Standards and Technology, Gaithersburg, MD 20899

**Keywords:** electron-impact cross sections, excitation, fitting formulas, helium, hydrogen, ionization

## Abstract

Compact fitting formulas, which contain four fitting constants, are presented for electron-impact excitation and ionization cross sections of atoms and ions. These formulas can fit experimental and theoretical cross sections remarkably well, when resonant structures are smoothed out, from threshold to high incident electron energies (< 10 keV), beyond which relativistic formulas are more appropriate. Examples of fitted cross sections for some atoms and ions are presented. The basic form of the formula is valid for both atoms and molecules.

## 1. Introduction

For applications in plasma modeling and the study of energy deposition in matter by charged particles, various analytic formulas have been used to fit ionization and excitation cross sections, *σ*, of neutral atoms, ions and molecules by electron impact. The Bethe theory [1,2] provides clear guidelines for choosing such analytic formulas at high incident energies, *E*, for electric dipole (E1) allowed transitions:

$$\sigma (x)=x^{-1}(a\;\ln\;x+b+c/x),\;\text{with}\;x=E/I,$$ (1)

where *I* is the ionization potential (or excitation energy for discrete excitations), and *a, b* and c are constants characteristic of the target but independent of *E*, or the scaled incident energy *x*.

The logarithmic term arises from the dipole interaction, and hence an El-forbidden transition will begin with the *b* term in Eq. (1). Actually, the expansion in Eq. (1) continues after the *c* term in negative powers of *x*. The Bethe formula is based on the first Born approximation. The physical effects not represented by the Born approximation — such as the distortion of the incident wave, polarization of the target charge distribution by the incident particle, and the electron exchange effect between the incident electron and bound electrons in the target—contribute terms between the *b* and *c* terms in Eq. (1). Hence, expanding the cross section formula beyond those included in Eq. (1) becomes a futile exercise unless the additional physical effects mentioned above are also included. The fitting formulas discussed in this article are only valid for E1-allowed transitions, since we keep the logarithmic term in our formulas.

According to the Mott scattering formula, which includes the electron exchange between the projectile and target electrons (or an ejected one in our case), the interference between the scattered and ejected electrons generated by electron-impact ionization leads to a term [2] of the form *x*^−2^ In *x* in *σ*(*x*). On the other hand, the distortion of the projectile wave function near the target nucleus introduces a term [3] of the form *x*^−1^×*σ*(plane-wave Born). Moreover, cross sections must vanish at the threshold, except for the discrete excitations of ions. The terms associated with In *x* vanish for El-forbidden transitions. Various fitting formulas [4,5] differ in the choice of functions to represent *σ* at low *x* toward the threshold at *x* = 1, and often contain more fitting constants than our formulas.

To accommodate the leading *x* dependence both of the electron exchange and the projectile-wave distortion mentioned earlier, it is desirable to introduce negative powers of *x* in the fitting formula. This was achieved by introducing (*x+D*) in the denominator with a fitting constant *D*, which provides additional flexibility. We found that the following compact formulas fit known cross sections remarkably well throughout the entire range of *I*⩽*E*⩽ 10 keV. For a cross section that vanishes at the threshold (*x* = 1), e.g., for the ionization of atoms, ions and molecules and for the excitations of neutral atoms and molecules, we use

σ(x)x≡S(x)=Alnx+[Blnx+C(x−1)]/(x+D), (2)

where *S* (*x*) is a scaled collision strength and *A, B, C*, and *D* are fitting constants. Cross sections for the E1-allowed excitations of ions do not vanish at thresholds, i.e., *σ*(*χ* = 1) > 0. In this case, we use

σ(x)x≡S(x)=Alnx+(Blnx+Cx)/(x+D). (3)

The scaled collision strength, *S* (*x*), monotonically increases with *x* in most cases, and the fitting constants are better determined using *S* rather than *σ* itself, particularly when the asymptotic behavior of the cross section at high *x* is known either through actual measurements or theories such as the Born approximation.

Often, electron-impact cross sections close to thresholds are crowded with resonances, mostly through the formation of transient multiply-excited states or negative ion states. Fitting formulas presented in this report are too simple to reproduce such resonances.

In Table 1, we list fitting constants [see Eq. (2)] that reproduce electron-impact ionization cross sections for some first-row atoms and ions. For the hydrogen atom, we fitted to the experimental ionization cross section measured by Shah, Elliott, and Gilbody [6]. For the helium atom, we used the experimental ionization cross section by Shah et al. [7] and the asymptotic Bethe cross sections by Kim and Inokuti [2]. For the ionization of He^+^ and Li^+^, the cross sections measured by Dolder and coworkers [8,9] and the asymptotic cross sections from Ref. [2] were used. Our fitting reproduces the original data within a few percent, except for minor local departures mostly near ionization thresholds.

**Fig. 1:**
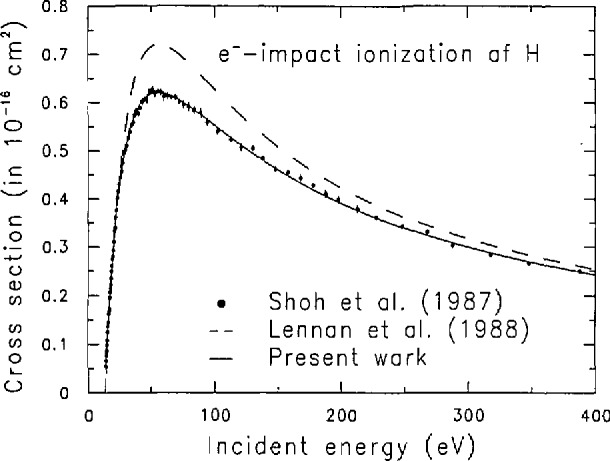
Cross section for the ionization of the hydrogen atom by electron impact. Solid circles represent the experiment by Shah, Elliott, and Gilbody [6], the dashed curve represents the cross seclion recommended by the Belfast group [5], and the solid curve is our filling to the experimental data [6].

We note that the ionization cross section of the hydrogen atom recommended in Ref. [5] is about 15% higher than the latest experimental value [6] at the peak cross section. These two cross sections are compared in Fig. 1.

**Fig. 2:**
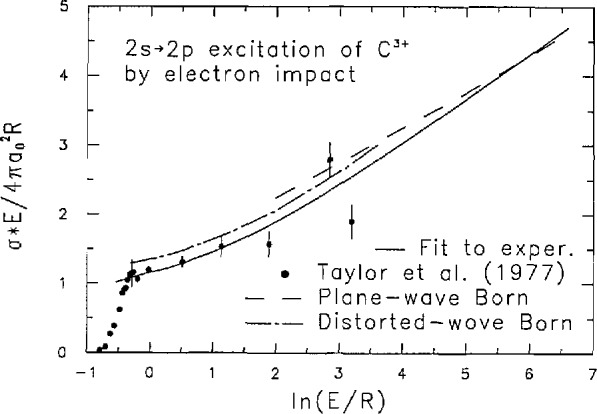
Collision strength for the 2*s* ^2^S→2*p* ^2^P excitation of C^3+^ by electron impact. The plane-wave and distorted-wave Born cross sections were calculated by the present author using Hartree-Fock wave functions. In the figure, *σ* is the cross section, *E* is the incident electron energy, *a*_0_ is the Bohr radius (5.29 nm), and *R* is the rydberg energy (13.6 eV).

As an example of El-allowed discrete excitations, fitting constants [see Eqs. (2) and (3)] for the 1*s* ^2 1^*S→* 1*snp* ^1^p(*n* = 2–4) excitations of He [10,11] and the 2s ^2^S→2*p* ^2^P excitation of C^3+^ by electron impact [12] are listed in Table 2. The He excitation cross sections were combined with the Bethe cross sections at high incident energies [13]. Also, the experimental data on the excitation of C^3+^ by Taylor et al. [12] were smoothly joined to the plane-wave Born cross section at high incident energies using the shape of the distorted-wave Born cross section. The Born cross sections were evaluated by the present author using Hartree-Fock wave functions for the ground and the *2p* ^2^P states as is shown in Fig. 2. Equation (2) should also be useful in fitting electron-impact cross sections for molecules.

To calculate the rate coefficients 〈*σν*〉 averaged over a Maxwellian distribution, we recommend that eight-point Laguerre quadrature be used, as suggested in Ref. [5]:

$$\langle \sigma v\rangle =6.692\times 10^{7}{\left(kT\right)}^{1/2}\sum_{i}\;\omega_{i}S(1+kT\;x_{i}/I),$$ (4)

where *kT* is the (plasma) electron temperature in eV, and *ω_i_*, and *x_i_*, are the Laguerre quadrature weights and abscissas, respectively.

Note that the integration formula (8) in Ref. [5] has a misprint: *x_i_* there should be replaced by (*x_i_ +I/kT*). Nevertheless, the rates reported in Ref. [5] are correct. The fitting constants in Tables 1 and 2 will lead to cross sections in 10^−16^ cm^2^ and collision rates in cm^3^/s.

For El-forbidden transitions, the logarithmic terms in the fitting formulas vanish. The remaining two terms with *C* and *D* coefficients alone do not provide enough flexibility to fit E1-forbidden cross sections except in unusually simple cases. Instead, a power series in (*x* −1),

$$\sigma x=\sum_{i}A_{i}{\left(x-1\right)}^{i},$$ (5)

where th *A_i_ are fitted coefficients (i*⩽*5)*, should be adequate to reproduce most E1-forbidden cross sections.

Also, the formulas presented here are not suitable for fitting proton-impact ionization cross sections at low incident energies, say *E*~300keV or lower, because the analytic form used here cannot account for ionization by charge transfer—an electron in the target attaching itself to the incident proton—which begins to dominate the ionization process at *E* ~300 keV and below.

## Figures and Tables

**Table 1:** The constants *I*, *A*, *B*, *C*, and *D* [see Eq. (2)] for electron-impact ionization of atoms and ions. The ionization potential *I* is given in eV, and the filling constants *A*, *B*, *C*, and *D* are in 10^−16^cm^2^

Atom	*I*(eV)	*A*	*B*	*−C*	*D*
H	13.61	0.7576	−5.521	5.867	2.948
He	24.58	0.7326	−5.117	3.295	2.468
He ^+^	54.42	0.06233	−0.2982	0.2835	0.327
Li ^+^	75.60	0.08329	−0.4476	0.3225	4.252

**Table 2:** The constants *I*, *A, B, C* and *D* [see Eq. (2)] for the 1*s ^2 1^*S→1*snp* ^1^P *(n* = 1–4) transition of He and the 2*s* ^2^S→2*p* ^2^P excitation of C^3+^ by electron impact. The excitation energy *I* is given in eV, and the fitting constants *A*, *B, C*, and *D* are in Å^2^

Atom	Excited state	*I*(eV)	*A*	*B*	*C*	*D*
He	1*s*2*p* ^1^P	21.22	0.3991	−0.3314	−0.00325	−0.012
He	1*s*3*p* ^1^P	23.09	0.08978	−0.0499	0.01003	−0.789
He	1*s*4*p* ^1^P	23.75	0.03488	−0.0286	0.00571	−0.343
C^3+^	2*p*^2^P	8.004	3.938	6.373	0.0061	−0.999
